# Left Atrial Strain—Current Review of Clinical Applications

**DOI:** 10.3390/diagnostics15111347

**Published:** 2025-05-27

**Authors:** Constantin Andrei Rusali, Ioana Caterina Lupu, Lavinia Maria Rusali, Lucia Cojocaru

**Affiliations:** 1 Department of Cardiology, Constanta County Clinical and Emergency Hospital, Ovidius University of Constanta, 145 Tomis Boulevard, 900591 Constanta, Constanta County, Romania; andrei1678@yahoo.com; 2 Department of Internal Medicine, Ovidius University of Constanta, 145 Tomis Boulevard, 900591 Constanta, Constanta County, Romania; caterinasrb@gmail.com (I.C.L.); lavinia567@yahoo.com (L.M.R.)

**Keywords:** left atrial strain, echocardiography, atrial function, heart failure, atrial fibrillation, cardiac imaging, prognostic marker

## Abstract

Left atrial strain has gained significant attention in recent years due to its potential to provide valuable insights into the function and mechanics of the left atrium. This review aims to evaluate the current applications of LA strain in clinical practice, particularly in assessing various cardiac conditions, including heart failure, atrial fibrillation, valvular heart disease, and coronary artery disease. We summarize the latest evidence regarding the role of left atrial strain in assessing left atrial remodeling, predicting outcomes, and its potential use as a prognostic tool. Unlike previous reviews focusing on single disease states, this review synthesizes emerging data across multiple cardiac conditions, highlighting novel implications for clinical practice. Left atrial strain emerges as a promising non-invasive marker for evaluating atrial function and guiding clinical decision-making. However, further research must fully establish its role across diverse patient populations and clinical settings.

## 1. Introduction

The left atrium (LA) is more than a simple passive conduit, playing an integral part in the cardiac cycle. The LA has three fundamental functions: reservoir, conduit, and contractile function.

Reservoir function: During the left ventricle (LV) systole, the LA is a reservoir for receiving blood from the pulmonary veins. This phase takes place during LV contraction and isovolumetric relaxation and depends on the compliance of the LA [1].

Conduit function: A passive phase in which the blood flows from LA to LV. This phase takes place during LV relaxation and diastasis and depends on the pressure gradient between LA and LV and the compliance of the LV [2].

Contractile function: this phase takes place during LV telediastole. The LA has an active contraction and acts as a pump, pushing approximately 20% of the blood flow to the LV. This pump function depends on preload, atrial myocardial contractility, and telediastolic LV pressure and thus becomes truly important when LV filling pressure is increased [3].

**Figure 1:**
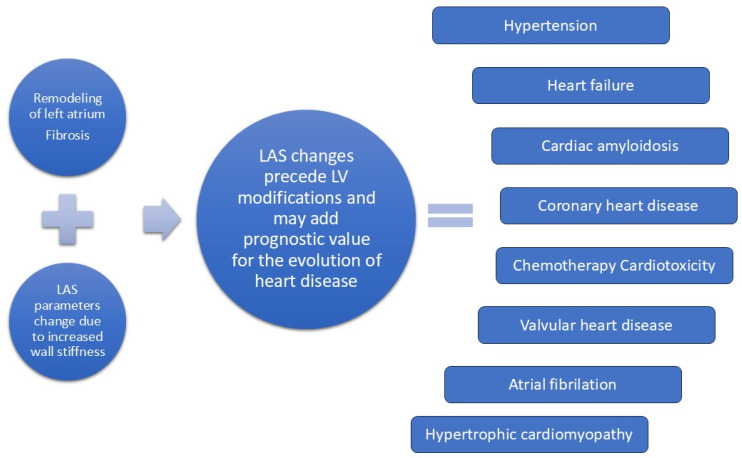
Use of left atrial strain.

Analyzing left atrium function can improve the diagnosis of subclinical left ventricle dysfunction and provide prognostic value for the evolution of various heart diseases (Figure 1).

## 2. Assessment of LA Strain (LAS) by Echocardiography

The LAS can be analyzed either through speckle tracking echocardiography (STE) or by using other imaging techniques such as cardiac magnetic resonance (CMR) or cardiac computed tomography (CCT). Several recent studies have compared the assessment of LAS by STE with that obtained by CMR and CCT [4,5,6,7], showing that STE remains the superior method due to its higher accessibility and reliability.

**Figure 2:**
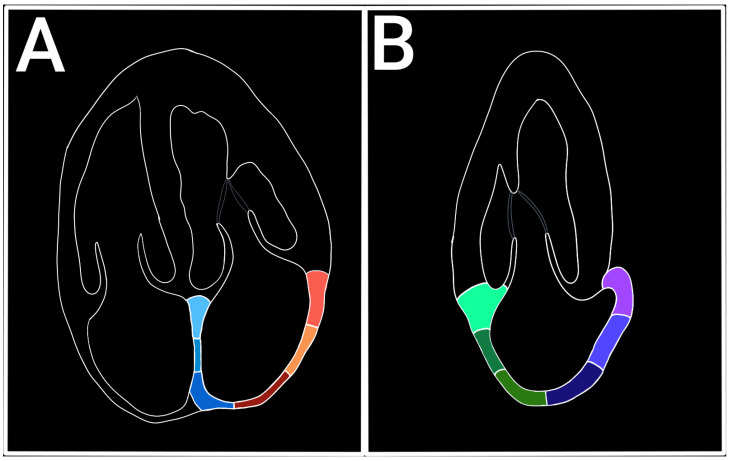
Segmentation of the left atrium. (**A**) Apical four chambers. (**B**) Apical two chambers.

LAS represents the longitudinal deformation of the LA myocardium and evaluates the atrial function independent of its volume, an early marker of atrial dysfunction [8,9]. The LAS has a positive value during the filling phase (reservoir) when the LA dilates and the distance between two atrial points (or speckles) increases, and a negative value during the contraction phase when the distance between two atrial points shortens. The views used for LAS measuring are the apical four-chamber view (A4C) and the apical two-chamber view (A2C). The A4C view is the gold standard for LAS evaluation, while the A2C view can be used to complete the assessment. For speckle tracking analysis, the A4C view must be zoomed in on the LA without losing the valvular landmarks, excluding the pulmonary veins and the LA appendix [10]. The optimal frame rate should be between 60 and 80 fps. A lower frame rate reduces the sensibility to the rapid movement of the LA myocardium [11] (Figure 2).

In order to precisely determine LAS through STE, it is crucial to synchronize the image with the ECG tracing. It is also recommended that the data be correlated with pulsed Doppler recording to establish better the beginning/ending of certain phases in the cardiac cycle. There are two possibilities for starting points in LAS measuring—the R wave and the P wave on ECG. Setting the starting point at the upslope of the R wave coincides with the telediastole, marking the closure of the mitral valve. This approach is more feasible, offers a higher reproducibility rate than the P wave [12], and permits a better analysis in patients with atrial fibrillation [13]. The downsides of this method are the possible overestimation of the reservoir and conduit phases and the possible omission of the negative curve associated with atrial contraction [13]. By choosing the P wave as a reference point, we mark the beginning of the atrial systole. The limitations of this method are: (1) it needs a high-quality ECG, which can be difficult in tachycardic rhythms, artifacts, or obese patients; and (2) it could omit the beginning of the reservoir phase. The benefits are: (1) it permits a detailed analysis of all three phases of the atrial cycle [14]; and (2) the possibility to evaluate the atrial contraction after conversion of atrial fibrillation or after paroxysmal atrial fibrillation. The ASE/EACVI guidelines recommend the upslope of the R wave as a gold standard for the reference point.

The evolution of the left atrial strain curve reflects the atrial dynamics during the cardiac cycle and consists of three phases:

The reservoir phase is between the closure and the opening of the mitral valve (concurs with ventricular contraction and isovolumetric relaxation). The LA acts as a passive reservoir and distends as it fills with blood from the pulmonary veins. During this phase, the atrial longitudinal strain increases to a positive peak at the end of the atrial filling (peak atrial longitudinal strain—PALS).

The conduit phase starts with the opening of the mitral valve and continues during the early ventricular diastole (E-wave). The atrial strain decreases with the rapid emptying of the LA until it reaches a plateau corresponding to the atrial diastasis.

The contraction phase (booster pump) starts in ventricular telediastole and consists of atrial contraction—it corresponds to the A-wave on pulsed Doppler. During this phase, the longitudinal strain continues to drop, reaching a minimum during atrial contraction—peak atrial contraction strain (PACS) [15].

If we base the analysis on the ventricular function, then the reference point will be the QRS complex. In this situation, the first positive wave corresponds to the reservoir function (LAS-r) and the descending curve—rapid filling and atrial contraction—corresponds to the conduit (LAS-cd) and pump function (LAS-ct), respectively [16].

**Figure 3:**
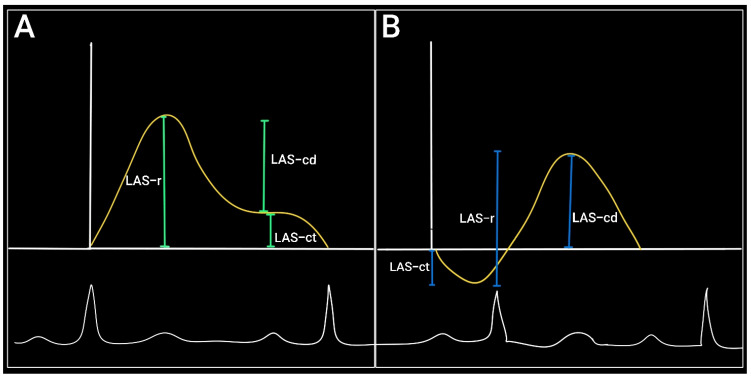
Left atrial strain assessment (yellow) (**A**) shows the QRS complex as the reference point. (**B**) shows the P wave as the reference point (ECG in white). LAS-r = reservoir function; LAS-ct = pump function; LAS-cd = conduction function.

If we choose the P wave as a reference point, the first negative peak will reflect the pump function, the positive peak will correspond to the conduit function, and the sum of the two will signify the reservoir function [17] (Figure 3).

The most recent EACVI consensus [18,19] establishes the end-diastole (R wave or the nadir of the atrial strain curve) as the primary reference point. This is because it is useful independent of the heart rate and also because it is easy to calculate LAS-r, which is the parameter with the most studied prognostic value [20,21,22,23,24].

STE also enables the determination of left atrial strain rate (LASR). It calculates the deformation rate of the atrial myocardium. If we choose the R wave as a reference point, the LASR curve will have one positive and two negative peaks. The positive peak corresponds to the reservoir phase during the ventricular systole (SRs) and two negative peaks—the first reflecting the early rapid ventricular filling—the conduit phase (SRe) and the second representing the atrial contraction (SRa) [25].

An important disadvantage of LASR is the difficulty of determining irregular rhythms such as atrial fibrillation due to the large variations in the cardiac cycle [26].

Also, determining LASR in tachycardic rhythms requires an increased frame rate [27,28].

### Normal Values of Left Atrial Strain

Parameters of left atrial function measured by two-dimensional speckle tracking echocardiography include reservoir, conduit, and contractile LAS. Reference values have been proposed in several studies and international consensuses. In general, reservoir LAS has the highest clinical value, reflecting the maximum extension of the atrium in ventricular systole, while conduit and contractile LAS assess passive and active atrial function. Normal LAS parameters are presented in Table 1 [29,30,31,32].

## 3. Left Atrial Strain in Hypertension

In recent years, LAS has been rising as a potent marker that can detect early subclinical changes in the left atrium that can precede structural modifications. Mondillo et al. investigated atrial function in individuals diagnosed with hypertension and diabetes with normal left atrial dimensions [33]. They reported that peak atrial longitudinal strain was significantly lower in patients with hypertension (29.0 ± 6.5%) and diabetes (24.7 ± 6.4%) compared to controls (39.6 ± 7.8%). The lowest values were observed in those with both hypertension and diabetes (18.3 ± 5.0%) (*p* < 0.0001). These findings indicate atrial dysfunction may manifest before observable structural changes occur.

Another 2022 study published by Taamallah et al. showed that left atrial longitudinal strain during the reservoir and conduit periods is impaired in patients with hypertension despite normal cavity size and before the detection of other echocardiographic changes [34].

Ting-Yan Xu et al. investigated 248 patients (124 with hypertension), demonstrating that hypertension is associated with impaired LA function, as assessed by STE strain imaging techniques, even before LA enlargement develops and after LV structural and functional remodeling is accounted for [35]. Furthermore, in the presence of impaired LA function, even white-coat or masked hypertension might be treated with hypertensive drugs.

In 2024, Stefani et al. conducted a study involving 208 patients with hypertension, comparing those without left ventricular hypertrophy (LVH) to controls [36]. They found that patients with non-LVH hypertension exhibited significantly lower left atrial reservoir strain (LAS-r) (29.78 ± 6.08 vs. 34.78 ± 29.78; *p* = 0.022) and conduit strain (LAS-cd) (14.23 ± 4.59 vs. 19.66 ± 7.29; *p* = 0.014) despite comparable left atrial volumes (LAV). However, left atrial contractile strain (LAS-ct) did not differ significantly between the two groups (15.12 ± 3.77 vs. 15.56 ± 3.79; *p* = 0.601).

A study from 2022 conducted on 290 hypertensive patients showed that left atrial stiffness index was significantly higher in non-dippers [0.29 (0.21, 0.41)] than in dippers [0.26 (0.21, 0.33)] (*p* < 0.05 Brachial-ankle pulse wave velocity to global longitudinal strain (GLS) ratios were notably higher in non-dippers [−80.9 (−69.3, −101.5)] compared to dippers [−74.2 (−60.2, −90.6)] (*p* < 0.05)). Additionally, non-dippers demonstrated significantly lower values for LAS-S, LAS-E, LAS-A, and LV GLS compared to dippers (*p* < 0.05) [37].

A 2021 meta-analysis comprising 17 studies with a total of 1723 participants—including 951 women with gestational hypertension (680 of whom were preeclamptic) and 772 controls—found that gestational hypertension was associated with greater cardiac maladaptation, as indicated by significantly lower GLS values compared to normal pregnancies [38].

A study published in 2024 by Mousa et al., which included patients recently diagnosed with systemic arterial hypertension, showed that PALS, PACS, E/e’, and the LA stiffness index improved in hypertensive patients with controlled blood pressure values [39].

Girard et al. conducted a small study involving 36 patients with resistant hypertension treated with spironolactone for 6 months. The study observed a non-significant increase in reservoir strain (29.1 ± 8.5% vs. 30.9 ± 5.5%; *p* = 0.068), while active strain showed a significant rise from baseline (16.3 ± 4.1% vs. 17.8 ± 4.2%; *p* < 0.05), independent of baseline aldosterone levels [40].

## 4. Left Atrial Strain and Heart Failure with Preserved Ejection Fraction

Heart failure with preserved ejection fraction (HFpEF) is defined in the 2021 ESC guidelines as (1) symptoms and signs of HF, (2) an LVEF ≥ 50%, and (3) objective evidence of cardiac structural and/or functional abnormalities consistent with the presence of LV diastolic dysfunction/raised LV filling pressures, including raised natriuretic peptides (NPs)**.** HFpEF emerges as a clinical entity with increasing prevalence, affecting mainly the elderly population, hypertensive patients, and those with multiple comorbidities. In HFpEF, the diastolic dysfunction of LV determines an increase in LV filling pressure, which in turn triggers the LA functional and structural remodeling [41].

In HFpEF the LA reservoir strain is mild to moderately reduced, the LA conduit strain is reduced (due to increased stiffness), and the LA pump strain is preserved in the initial phases and reduced in later stages [42].

Table 2 provides a selection of recent studies regarding LAS in HFpEF [42,43,44,45,46]:

## 5. Left Atrial Strain in Heart Failure with Mildly Reduced Ejection Fraction

Heart failure with mildly reduced ejection fraction (HFmrEF) (LVEF = 40–49%) was introduced as a separate clinical entity in the 2016 ESC guidelines. This particular type of HF has intermediary characteristics between those of HFrEF and HFpEF. The prognosis for HFmrEF is better than HFrEF, almost similar to that of HFpEF [47].

An integral part of the pathogenic processes in chronic heart failure, irrespective of ejection fraction, is the increase of diastolic pressures of the LA. Increased diastolic pressure determines the stretching of the atrial myocardium and the activation of fibrotic processes. In time, the LA suffers a remodeling process, represented by dilation and fibrosis, with a progressive decrease in atrial compliance, which determines the increase of ventricular filling pressures, creating a vicious cycle. This pathogenic process is generically named atrial cardiomyopathy and includes all the complex changes in the atria’s morphology, mechanics, and electrophysiology [48].

LAS strongly correlated with all these modifications as a marker of atrial deformation.

An important concept is that LA dysfunction is a direct contributor to HF symptomatology. Normally, during physical effort, LV filling is improved by an increase in the reservoir and contractile functions of the LA. In patients with HFpEF, HFmrEF, and HFrEF, this functional reserve is compromised; thus, the effort tolerance decreases [49,50,51].

In Table 3 we included all the recent studies regarding the role of LAS determination in HFmrEF [22,52,53,54].

## 6. Left Atrial Strain in Heart Failure with Reduced Ejection Fraction

In patients with heart failure with reduced ejection fraction (HFrEF), high left atrial pressure (LAP) is a frequent finding and can signify disease progression or decompensation of heart failure [55].

LAS, particularly LASR, has become an important echocardiographic parameter in evaluating patients with HFrEF. LASR significantly correlates with left atrial and LV filling pressure and is a valuable alternative to current ASA/EACVI algorithms for estimating LV diastolic dysfunction [56].

E/A ratio, for example, is often unavailable or significantly affected by atrial arrhythmias (e.g., atrial fibrillation) and/or mitral valvopathies. E/A ratio is influenced by moderate/severe mitral regurgitation or stenosis, with an elevation of E wave velocity and LA dilation, making the evaluation of LAP by this algorithm unreliable. In atrial fibrillation, the A wave (corresponding to atrial contraction) is missing, and the LA is enlarged independent of LAP [57,58].

Although atrial dysfunction was considered to be more prevalent in HFpEF, recent studies show that patients with HFrEF have more severe atrial myopathy with lower LASR values. These findings suggest an intrinsic atrial myopathy influenced by systolic ventricular dysfunction and functional mitral regurgitation [59].

Table 4 contains recent studies into the use of LAS in HFrEF [22,55,59,60,61,62,63,64,65,66]:

## 7. Left Atrial Strain in Cardiac Amyloidosis

Cardiac amyloidosis (CA) is a particular form of restrictive infiltrative cardiomyopathy characterized by extracellular deposits of abnormally folded amyloid fibrils in the myocardium. This deposition results in increased stiffness of the ventricular myocardium with a progressive thickening that determines diastolic dysfunction, elevated filling pressure of the LV, and finally, LA enlargement and dysfunction [63].

In 2023, Monte et al. showed a significant reduction in all three LAS phases in patients with CA compared with those with hypertrophic cardiomyopathy and the control group. These modifications were present even in patients with CA and preserved ejection fraction, highlighting the early atrial dysfunction in CA [64].

LAS proved useful in the differential diagnosis between CA and other forms of ventricular hypertrophy, such as Fabry disease. In a study from 2024, Matting et al. demonstrated that LAS can provide an accurate differential diagnosis between CA and Fabry disease, with a sensitivity of 90% and specificity of 64% for a reservoir LAS of 20% [65].

Table 5 contains recent studies into the use of LAS in cardiac amyloidosis [66,67,68,69,70].

## 8. Left Atrial Strain and Coronary Heart Disease

It is a well-known fact that coronary artery disease is responsible for one-third of global deaths, and thus, identifying new prognostic parameters for short- and long-term evolution after coronary syndrome is a continuous task [71].

Although risk assessment tools can be valuable aids for physicians in establishing a patient therapeutic plan, new parameters are continuously needed to prevent better and timely initiate therapy in patients at risk or with coronary artery disease [72].

In 2024, Pedersson et al. investigated the long-term prognostic value of left atrial strain indices—PALS and PACS—as prognostic factors for all-cause mortality in patients with acute coronary syndromes [73]. PALS and PACS proved to be independent predictors of mortality. A decrease of 1% in PALS was associated with a high risk of death (HR 1.04, *p* = 0.002). This association was observed even in patients with a normal LA volume index (LAVI) [73].

In ST-elevation myocardial infarction patients, LAS was an independent predictor for heart failure. A study by Ricken et al. published in 2024, with a mean follow-up period of 8.8 years, showed that LASR has incremental value in the prediction of cardiovascular death, hospitalization for heart failure, and new onset heart failure when added to the prediction model comprised of age, sex, diastolic blood pressure, and left ventricular ejection fraction [74].

LASR also proved to be an independent prognostic factor for cardiovascular death and heart failure in 501 patients with acute myocardial infarction with or without atrial fibrillation in a study by Tangen et al. from 2024 and Sikora et al. from 2025 [75,76].

In a 2024 study that included patients with coronary artery disease, Tu et al. demonstrated that LASR combined with E/e’ ratio was an excellent predictor of elevated left ventricular filling pressures in patients with CAD. This combination had a stronger correlation with filling pressures than each parameter [77].

## 9. Left Atrial Strain in Atrial Fibrillation

Atrial fibrillation (AF) is the most prevalent sustained cardiac arrhythmia globally and is associated with increased morbidity and mortality. Early detection and risk stratification are paramount for effective management. AF disrupts the reservoir, conduit, and contractile phases of LA function, leading to structural and electrical remodeling [78]. Reduced LASR has been associated with elevated LA pressures and fibrosis, serving as a potential marker for AF onset, progression, and recurrence [78].

Over the past decade, numerous studies have explored the predictive value of LAS for elevated atrial pressure, new-onset atrial fibrillation (in various clinical settings, including the general population, post-stroke, post-myocardial infarction, and post-cardiac surgery), arrhythmia recurrence after cardioversion or ablation, and risk of thrombus formation (particularly in the LA appendage). Table 6 summarizes the most recent studies, highlighting key findings on this topic:

While LAS provides valuable insights into LA function with significant implications in detecting, managing, and prognostic AF, standardization of measurement techniques and reference values is needed. Further large-scale, prospective studies are warranted to validate LAS as a routine clinical tool in AF management with applicability in risk stratification and therapeutic decisions.

## 10. Left Atrial Strain in the Assessment of Chemotherapy-Induced Cardiotoxicity

Early diagnosis and improved cancer management have increased cancer survival [93], and thus, cardiotoxicity associated with antineoplastic treatments has become more prevalent, inducing both short- and long-term complications [94]. In this context, early detection of cardiac dysfunction is crucial to prevent irreversible damage.

Early detection of cardiotoxicity focuses on LV systolic function, with LV ejection fraction assessed by transthoracic echocardiography (TTE) being the most widely used method [95]. However, a reduced LV ejection fraction is a late manifestation of cardiotoxicity [96]. More recently, subclinical LV systolic dysfunction has been identified by LV global longitudinal strain (LV GLS) by 2D-STE [97]. Neither LV ejection fraction nor LV GLS can identify diastolic dysfunction, which is an early marker of chemotherapy-related cardiotoxicity that often precedes the onset of systolic impairment [98]. LAS has emerged as a sensitive and early marker of diastolic disfunction [99], thus, its usefulness in the early detection of cardiotoxicity is compelling. Reduced LAS, particularly in the reservoir phase, may indicate early diastolic dysfunction and elevated filling pressures as an early marker of cardiotoxicity. Already, several prospective studies have shown a decline in LAS-r and LAS-cd shortly after initiation of cardiotoxic chemotherapy (e.g., anthracyclines, trastuzumab), often preceding changes in LV GLS and LVEF [100,101,102,103,104,105]. Regular assessment of LAS during chemotherapy can help track evolving myocardial changes and guide timely intervention.

Also, LAS may help identify patients with subclinical cardiac dysfunction before starting cardiotoxic chemotherapy, thus identifying patients at higher risk for cardiotoxicity. Persistent LAS abnormalities post-therapy may indicate ongoing subclinical dysfunction and the necessity to continue monitoring. Integrating LAS into existing monitoring strategies could improve risk stratification and individualize cardio-oncology care, potentially leading to earlier interventions and improved patient outcomes.

Several studies have demonstrated the clinical utility of LAS in monitoring patients undergoing chemotherapy. Table 7 summarizes the most relevant publications from the past decade:

While short-term studies show promise, longer follow-up data are needed to correlate LAS changes with clinical outcomes. Also, there is still no universally accepted threshold for abnormal LAS in chemotherapy-induced cardiotoxicity and further research is needed to standardize measurement techniques and validate its prognostic utility across diverse patient populations.

## 11. LAS in Valvular Heart Disease

Valvular heart diseases (VHD), such as mitral regurgitation, mitral stenosis, and aortic stenosis, impose significant hemodynamic burdens on the LA. Chronic pressure and volume overload lead to structural remodeling and functional impairment of the LA, which are pivotal in the progression of VHD and its complications.

### 11.1. LAS in Mitral Regurgitation (MR)

In MR, the LA is subjected to volume overload, leading to dilation and functional deterioration. In a study of 121 patients with severe MR, Philippe Debonnaire et al. demonstrated that LAS-r was an independent predictor (odds ratio, 0.88; 95% confidence interval, 0.82–0.94; *p* < 0.001), which had the highest accuracy in identifying patients with indications for mitral surgery. Patients with a left atrial reservoir strain (LAS-r) of 24% or lower had significantly reduced survival following mitral valve surgery, with a median follow-up of 6.4 years (*p* = 0.02), irrespective of their preoperative symptoms. Adding LAS-r to conventional surgical criteria enhanced the prediction of postoperative outcomes [107]. Separately, a study by Leen van Garsse et al. demonstrated that left atrial strain may aid in identifying individuals with chronic ischemic mitral regurgitation who are unlikely to derive benefit from undersized mitral ring annuloplasty. Specifically, lower values of left atrial peak global strain, peak systolic strain rate, and peak early diastolic strain rate were associated with recurrent MR after surgery [108]. Another possible applicability of LAS in patients with MR is the prediction of cardiovascular outcomes in patients with moderate asymptomatic MR to identify higher-risk groups and, therefore, the optimal time of surgery. Matteo Cameli et al. studied 395 patients with primary degenerative moderate asymptomatic MR. They demonstrated that patients who developed cardiovascular events presented reduced PALS, reduced LA emptying fraction, larger LAVI, and lower LV strain at baseline. From those, global PALS < 35% showed the greatest predictive performance to optimize the timing of surgery before the development of irreversible myocardial dysfunction [109].

### 11.2. LAS in Mitral Stenosis (MS)

MS results in increased LA pressure and subsequent remodeling. STE-derived LAS parameters, including reservoir, conduit, and contractile strains, are markedly diminished in patients with severe MS. These reductions correlate with the severity of stenosis and pulmonary hypertension, underscoring the role of LAS in assessing disease burden and guiding therapeutic decisions [110].

### 11.3. LAS in Aortic Stenosis (AS)

In 2023, Tan et al. showed that PALS independently predicts major cardiovascular events, having a higher predictive value than other conventional echocardiographic parameters. LAScd < 6%, LASr < 20%, and LASct < 12% were associated with a higher probability of cardiovascular events [111].

A systematic review of 18 studies (2660 patients) confirmed that a decreased PALS is associated with an increased risk of adverse events (death, heart failure, need for valve replacement), a 1% decrease in atrial strain being linked to a significant increase in risk [112].

Sonaglioni et al. (2021) demonstrated that a reduced atrial reservoir strain (LASr < 19%) in patients with moderate and asymptomatic aortic stenosis identifies a subgroup with unfavorable outcomes, in whom early intervention or more careful monitoring could be beneficial [113].

It has been observed that after pressure overload is removed by transcatheter valve implantation, atrial function (measured as LAS) improves significantly in the first months post-procedure, often before the visible reduction in atrial size [114].

Atrial strain recovery can provide information about reverse cardiac remodeling: patients in whom atrial strain remains severely depressed post-procedure or does not improve have a higher incidence of adverse events (e.g., heart failure or new-onset atrial fibrillation) in the short- and medium-term follow-up [115].

## 12. Method Limitations

Despite its considerable clinical potential, using left atrial strain (LAS) still presents several limitations that prevent its widespread adoption in routine cardiology practice. One of the main constraints is the lack of clear standardization of acquisition and analysis techniques, with significant variations between different echocardiographic platforms and between operators. In addition, the reproducibility of measurements can be influenced by examiner experience, image quality, and individual anatomical factors. Another major limitation is the absence of universally accepted reference values, which reduces the comparability of results between populations and centers. LAS is also affected by extracardiac factors, such as systemic comorbidities or left ventricular dysfunction, which can independently alter left atrial function. Finally, although LAS has demonstrated its prognostic value in numerous clinical settings, its practical utility remains to be validated, and extensive prospective studies are needed to define its role clearly in current diagnostic and therapeutic algorithms.

## 13. Conclusions

Current evidence supports the utility of LAS in predicting adverse cardiovascular events, risk stratification, and early detection of atrial dysfunction in multiple clinical conditions, such as heart failure with preserved ejection fraction (HFpEF), atrial fibrillation, cardiomyopathies, and valvular diseases. LAS also shows promising potential in guiding therapeutic strategies and monitoring response to treatment. Larger studies are still needed to implement this method in daily clinical practice.

## Figures and Tables

**Table 1:** Normal values of left atrial strain.

Study/Guideline	Population	Reservoir LAS (Mean ± SD)	LAS Conduit (Mean± SD)	LAS Contractile (Mean ± SD)
Sun et al. (2020) [29]	Healthy adults (Korea)	35.9 ± 10.6%	21.9 ± 9.3%	13.9% ± 3.6%
Pathan et al. (2017) [31]	Meta-analysis (21 studies)	40 ± 8%	27 ± 8%	15 ± 6%
Yafasov et al. (2024) [32]	Copenhagen	Abnormal thresholds		
		18.4%	6.8%	22.2%
Sugimoto et al. (2018) [30]	European	Mean ± SD or medial (IQR)		
		42.5 (36.1 to 48.0)	25.7 (20.4 to 31.8)	16.3 (12.9 to 19.5)

IQR, interquartile range; SD, standard deviation.

**Table 2:** Recent studies regarding LAS in HFpEF.

Year	Title	No. of Patients	Main Conclusions
2024	Impaired Left Atrial Reserve Function in Heart Failure With Preserved Ejection Fraction [43]	240	LA reservoir function is reduced in patients with HFpEF and it correlates with effort tolerance
2023	Prognostic Implications of Left Atrial Stiffness Index in Heart Failure Patients With Preserved Ejection Fraction [42]	307	An increased atrial stiffness index is associated with higher mortality risk and number of hospitalization in patients with HfpRF
2022	Left Atrial Strain Predicts Exercise Capacity in Heart Failure Independently of Left Ventricular Ejection Fraction [44]	171	A reduced LA reservoir strain is independently associated with a significantly reduced effort capacity
2021	Prognostic Value of Minimal Left Atrial Volume in Heart Failure With Preserved Ejection Fraction [45]	347	The minimum LA volume is a stronger predictor for cardiovascular effect compared with the maximum indexed volume
2020	Left Atrial Strain in Evaluation of Heart Failure with Preserved Ejection Fraction [46]	450	LASR could identify patients with HFpEF and increased filling pressures in the absence of an effort test

**Table 3:** Recent studies regarding LAS in HFmrEF.

Title	Authors	Paper	Year	No. of Subjects	Main Conclusions
Left Atrial Function in HFmrEF Differs from that of HFpEF [52]	Al Saikhan et al.	Eur Heart J Cardiovasc Imaging	2019	184	LAS (PALS and PACS) is significantly lower in HFmrEF compared with HFpEF, indicating an intrinsic atrial disfunction.
Prognostic Value of Left Atrial Strain in Heart Failure [22]	Jia et al.	Front Cardiovasc Med	2022	7787	Peak atrial longitudinal strain was identified as an independent predictor of all-cause mortality and cardiac hospitalization across all heart failure subtypes.
Left Atrial Function and Maximal Exercise Capacity in HFpEF and HFmrEF [53]	Maffeis et al.	ESC Heart Failure	2021	56	A reduced LAS (LA reservoir strain) correlates with low decreased exercise capacity in patients with HFmrEF and HFpEF.
Impact of SGLT2 Inhibitors on Left Atrial Functions in T2DM and HFmrEF [54]	El-Saied et al.	Int J Cardiol Heart Vasc	2023	45	SGLT2i significantly increase LAS (LAS reservoir, conduit, and contractile) in patients with type 2 diabetes and HFmrEF.

**Table 4:** Recent studies regarding LAS in HFrEF.

Title	Authors	Publication	Year	No. of Subjects	Main Conclusions
Prognostic Value of Left Atrial Strain in Heart Failure: A Systematic Review and Meta-Analysis [22]	Jia et al.	Frontiers in Cardiovascular Medicine	2022	7.787	LASR is an independent predictor of mortality and hospitalization rate in heart failure patients.
Left Atrial Reservoir Strain as a Predictor of Cardiac Outcome in Patients with Heart Failure: The HaFaC Cohort Study [60]	Bouwmeester et al.	BMC Cardiovascular Disorders	2022	1.000+	A reduced LASR is associated with an increased risk of cardiovascular events in patients with HFrEF.
Left Atrial Structure and Function in Heart Failure with Reduced (HFrEF) Versus Preserved Ejection Fraction (HFpEF) [55]	Jin et al.	Heart Failure Reviews	2021	8.806	Atrial disfunction is more severe in HFrEF, comparative with HFpEF, being a sign for intrinsic atrial myopathy.
Prognostic Power of Left Atrial Strain in Patients with Acute Heart Failure [61]	Park et al.	European Heart Journal—Cardiovascular Imaging	2021	1.000+	LASR >16% at discharge is associated with better survival rate without major cardiac adverse effects.
Left Atrial Strain in the Assessment of Diastolic Function in Heart Failure [62]	Carluccio et al.	Circulation: Cardiovascular Imaging	2023	864	PALS improves diastolic disfunction classification and the prediction of acute events in patients with heart failure.
Patients with Chronic Heart Failure and Predominant Left Atrial Versus Left Ventricular Myopathy [59]	Jin et al.	Cardiovasc Ultrasound	2025	374	Most heart failure patients exhibit balanced LA/LV myopathy; however, those with predominant LA myopathy tend to be older, have higher rates of atrial fibrillation and diabetes, elevated cardiac stress and injury biomarkers, and poorer clinical outcomes.

**Table 5:** Recent studies regarding LAS in cardiac amyloidosis.

Title	Authors	Publication	Year	No. of Subjects	Main Conclusions
Left Atrial and Left Ventricular Deformation in Identifying Advanced Stage of Cardiac Amyloidosis [66]	Puwanant et al.	JACC	2023	50	LASR was significantly reduced in patients with advanced stage of cardiac amyloidosis than in patients with less advanced stage, and showed high diagnostic accuracy in discriminating advanced stage cardiac amyloidosis
Prognostic Value of Left Atrial Strain in Patients with Wild-Type Transthyretin Amyloid Cardiomyopathy [67]	Oike et al.	ESC Heart Fail	2021	129	A higher LASR is associated with a significantly higher probability of total cardiovascular death and rate of heart failure related hospitalizations in patients with ATTRwt-CM even after adjusting for conventional predictive factors
Assessing Left Atrial Dysfunction in Cardiac Amyloidosis using LA-LV Strain Slope [68]	Edbom et al.	Eur Heart J Imaging Methods Pract	2024	59	PALS demonstrates an independent ability to differentiate ATTR-CM from LVH
Clinical Importance of Left Atrial Infiltration in Cardiac Transthyretin Amyloidosis [69]	Bandera et al.	JACC	2022	906	In patients with cardiac transthyretin amyloidosis, atrial electromechanical dissociation was associated with poorer prognosis compared with subjects in sinus rhythm and effective mechanical contraction
Left Atrial Mechanics and Left Ventricular Function in Cardiac Amyloidosis Patients Treated with Tafamidis [70]	Carvalheiro et al.	European Heart Journal—Cardiovascular Imaging	2025	28	After 1 year of treatment with tafamidis there was a significant reduction of all three phases of LAS

**Table 6:** Recent studies regarding LAS in atrial fibrilation.

Year	Authors	Study Title	Number of Patients	Main Conclusions
LAS in evaluating left atrial pressure (LAP) and function in AF				
2024	Xu Y et al. [79]	Left Atrial Strain Parameters to Predicting Elevated Left Atrial Pressure in Patients with Atrial Fibrillation	142	LASR, LA stiffness index, and LA filling index were independently associated with elevated LAP. In patients with AF, LAS parameters could be useful to predict elevated LAP and non-inferior to conventional echocardiographic parameters.
2021	Uziębło-Życzkowska B et al. [80]	Correlations Between Left Atrial Strain and Left Atrial Pressures Values in Patients Undergoing Atrial Fibrillation Ablation	172	LASR correlates with invasively measured LA pressures; lower LASR is associated with higher pressures. LASR may serve as a reliable, non-invasive marker of LAP in AF.
LAS in predicting new-onset AF				
2025	Granchietti AG et al. [81]	Left Atrial Strain and Risk of Atrial Fibrillation after Coronary Artery Bypass-grafting	100	Low preoperative LAS-ct and LASR are significant predictors of postoperative AF in CABG patients.
2024	Beyls et al. [82]	Left Atrial Strain Analysis and New-onset Atrial Fibrillation in Patients with ST-segment Elevation Myocardial Infarction (STEMI)	175	All LAS parameters were significantly impaired in new-onset AF patients, especially LASR. LASR < 27% is significantly associated with increased risk of new-onset AF during hospitalization for STEMI, with high sensitivity and specificity.
2023	Serenelli M et al. [78]	Atrial Longitudinal Strain Predicts New-Onset Atrial Fibrillation: A Systematic Review and Meta-Analysis	N/A	Reduced LASR is a significant predictor of new-onset AF, suggesting its utility in early detection and prevention strategies.
2022	Svartstein AW et al. [20]	Predictive Value of Left Atrial Strain in Relation to Atrial Fibrillation Following Acute Myocardial Infarction	392	Lower LASR is an independent predictor of incident AF following STEMI, highlighting its prognostic value in post-MI patients.
2021	Hauser R et al. [83]	Left Atrial Strain Predicts Incident Atrial Fibrillation in the General Population: the Copenhagen City Heart Study	4466	Lower peak atrial longitudinal strain (PALS) and peak atrial contraction strain (PACS) independently predict incident AF in the general population, even among individuals with normal LA size and left ventricle function.
2019	Rasmussen SMA et al. [84]	Utility of Left Atrial Strain for Predicting Atrial Fibrillation Following Ischemic Stroke	186	Reduced LASR is associated with higher risk of developing AF after ischemic stroke, suggesting its utility in post-stroke AF risk assessment.
LAS in predicting AF recurrence after cardioversion and ablation				
2024	Zeng D et al. [85]	The Utility of Speckle Tracking Echocardiographic Parameters in Predicting Atrial Fibrillation Recurrence After Catheter Ablation in Patients with Non-Valvular Atrial Fibrillation	380	Lower LASR and higher LA stiffness were independent predictors of AF recurrence after catheter ablation. LASR ≤ 24.3% and LA stiffness > 0.55 were associated with higher recurrence rates.
2023	Sabanovic-Bajramovic N et al. [86]	Left Atrial Strain Significance in Prediction of Early Atrial Fibrillation Recurrence after Cardioversion and Ablation	94	Peak atrial longitudinal strain (PALS) ≤ 15% was an independent predictor of early AF recurrence after cardioversion or ablation, even in patients with non-dilated LA.
2022	Li Y et al. [87]	Left Atrial Strain for Predicting Recurrence in Patients with Non-Valvular Atrial Fibrillation After Catheter Ablation	95	Decreased LAS-ct independently predicts AF recurrence post-ablation; LAS may contribute to the risk stratification for AF patients and selection of suitable patients for catheter ablation.
2019	Moreno-Ruiz LA et al. [88]	Left Atrial Longitudinal Strain by Speckle Tracking as Independent Predictor of Recurrence after Electrical Cardioversion in Persistent and Long-standing Persistent Non-valvular Atrial Fibrillation	131	Lower global peak atrial longitudinal strain (GPALS) was significantly associated with AF recurrence at 6 months post-electrical cardioversion. GPALS ≤ 10.75% was an independent predictor of recurrence.
LAS in assessing thrombotic risk in AF and detecting LA appendage thrombus				
2023	Abdelhamid S et al. [89]	Association of Left Atrial Deformation Analysis by Speckle Tracking Echocardiography with Left Atrial Appendage Thrombus in Patients with Primary Valvular Heart Disease	200	Peak atrial longitudinal strain (PALS) < 10.5% was a significant predictor of LA appendage thrombus in patients with primary valvular heart disease, regardless of rhythm.
2017	Cameli M et al. [90]	Left Atrial Strain Predicts Pro-Thrombotic State in Patients with Non-Valvular Atrial Fibrillation	79	Global peak atrial longitudinal strain (PALS) < 8.1% was a strong predictor of LAA thrombus and/or reduced LAA emptying velocity, with high sensitivity and specificity.
2017	Kupczynska K et al. [91]	Association Between Left Atrial Function Assessed by Speckle-Tracking Echocardiography and the Presence of Left Atrial Appendage Thrombus in Patients with Atrial Fibrillation	87	Impaired LAS is associated with higher risk of LA appendage thrombus presence in AF patients, providing incremental value over the CH_2_ADS_2_-VASc Score.
2014	Sasaki S et al. [92]	Left Atrial Strain as Evaluated by Two-Dimensional Speckle Tracking Predicts Left Atrial Appendage Dysfunction in Patients with Acute Ischemic Stroke	120	Decreased LA peak systolic strain was independently associated with LA appendage dysfunction in patients with acute ischemic stroke. LA peak systolic strain < 19% predicted LA appendage dysfunction with high sensitivity and specificity.

**Table 7:** Recent studies regarding LAS in chemotherapy-induced cardiotoxicity.

Year	Authors	Title	Number of Patients	Main Conclusions
2024	Emerson P et al. [100]	Alterations in Left Atrial Strain in Breast Cancer Patients Immediately Post Anthracycline Exposure	128	LA strain is a promising marker of early diastolic dysfunction. Reduction in LASR demonstrated increased sensitivity as a potential marker of cardiotoxicity compared to reduction in LV GLS.
2024	Inoue K et al. [101]	Early Detection and Prediction of Anthracycline-Induced Cardiotoxicity	383	Patients who developed cardiotoxicity had greater reductions in LAS-r and LAS-ct 3 months after initiating anthracyclines. Early decline in LAS-r was independently associated with subsequent cardiotoxicity.
2024	Goyal A et al. [106]	Left Atrial Strain as a Predictor of Early Anthracycline-Induced Chemotherapy-Related Cardiac Dysfunction: A Pilot Systematic Review and Meta-Analysis	297	Anthracycline therapy significantly reduced LAS-r and LAS-cd. LAS may serve as an early indicator of cardiotoxicity.
2023	Di Lisi et al. [102]	Atrial Strain Assessment for the Early Detection of Cancer Therapy-Related Cardiac Dysfunction in Breast Cancer Women (The STRANO STUDY: Atrial Strain in Cardio-Oncology)	169	LAS parameters (PALS and LA stiffness) significantly changed during chemotherapy, correlating with GLS changes. A PALS variation > 20.8% identified patients likely to develop asymptomatic mild cardiotoxicity.
2023	Chen J et al. [103]	Assessment of Left Heart Dysfunction to Predict Doxorubicin Cardiotoxicity in Children with Lymphoma	23	LAS-r and LAS-cd decreased significantly after chemotherapy, correlating with LV GLS. LAS can be used for early detection of cardiotoxicity in pediatric lymphoma patients.
2021	Laufer-Perl M et al. [104]	Left Atrial Strain Changes in Patients with Breast Cancer During Anthracycline Therapy	40	LAS-r and LAS-cd reduction occur early during anthracycline therapy, showing significant correlation to the routine echocardiographic diastolic parameters, which may imply a role in the detection of early cardiotoxicity.
2018	Meloche J et al. [105]	Temporal Changes in Left Atrial Function in Women with HER2+ Breast Cancer Receiving Sequential Anthracyclines and Trastuzumab Therapy	51	Intrinsic LA compliance and contractile properties were reduced early with cancer therapy. Patients who developed cardiotoxicity had lower baseline LAS-r and LAS-cd.

## Data Availability

The data underlying this article will be shared on reasonable request to the corresponding author.

## Abbreviations

The following abbreviations are used in this manuscript:

A2C	Apical two chamber view
A4C	Apical four chamber view
AF	Atrial fibrillation
CA	Cardiac amyloidosis
CCT	Cardiac computed tomography
CMR	Cardiac magnetic resonance
DOAJ	Directory of open access journals
GPALS	Global peak atrial longitudinal strain
HFmrEF	Heart failure with mildly reduced ejection fraction
HFpEF	Heart failure with preserved ejection fraction
HFrEF	Heart failure with reduced ejection fraction
LA	Left atrium
LAP	Left atrial pressure
LAS	Left atrium strain
LAS-cd	Conduit function of LA
LAS-ct	Pump function of LA
LASR	Left atrial strain rate
LAS-r	Reservoir function of LA
LAV	Left atrial volume
LAVI	Left atrial volume index
LV	Left ventricle
LVEF	Left ventricle ejection fraction
LVGLS	Left ventricle global longitudinal strain
MDPI	Multidisciplinary Digital Publishing Institute
NP	Natriuretic peptides
PACS	Peak atrial contraction strain
PALS	Peak atrial longitudinal strain
STE	Speckle tracking echocardiography
STEMI	ST-segment elevation myocardial infarction
TTE	Transthoracic echocardiography
VHD	Valvular heart disease
